# One-Dimensional Croconate-Based Fe-CP as a High-Performance Anode Material for Lithium–Ion Batteries

**DOI:** 10.3390/polym15183728

**Published:** 2023-09-11

**Authors:** Lin Zhang, Xiaofei Zhang, Yingcai Gui

**Affiliations:** Henan Key Laboratory of Functional Salt Materials, Center for Advanced Materials Research, Zhongyuan University of Technology, Zhengzhou 450007, China; 202215064320@zut.edu.cn (X.Z.); 16692396319@163.com (Y.G.)

**Keywords:** coordination polymers, one-dimensional, croconate-based, anode

## Abstract

Coordination polymers (CPs) have attracted greater scientific attention as promising electrode materials for lithium–ion batteries (LIBs) due to their diverse and versatile structural chemistry. This study introduces a croconate-based one-dimensional CP, namely [Fe(C_5_O_5_)(H_2_O)_3_]_n_) (referred to as Fe-CP), as an efficient anode material with high-performance characteristics for rechargeable LIBs. The ligand with abundant redox sites coordinating to the transition metal ion endowed the anode material with a remarkable stability in the electrolyte, in addition to high capacity, high-rate capability, and high cycling performance during charging/discharging process. The Fe-CP has a unique chain-based supramolecular structure, setting it apart from other porous three-dimensional molecular materials. The presence of unrestricted channels between the chains facilitates the diffusion of lithium ions in this unique structure. When tested at 100 mA g^−1^ over a range of voltages between 0.01 and 2.4 V, the Fe-CP anode demonstrated a noteworthy specific capacity of 521 mA h g^−1^ over 140 cycles. Moreover, the Fe-CP anode material exhibited excellent rate performance and demonstrated favorable cyclability. Following exposure to high charging and discharging rates of 2 A g^−1^, the anode ultimately regained its initial capability when the current rate was back at 100 mA g^−1^.

## 1. Introduction

Along with the substantial development of economic globalization, non-renewable energy such as fossil oil, coal, and natural gas are becoming scarce due to overuse. Climate change and environmental protection have become critical problems to be solved in the new era. The pursuit of green and renewable energy sources such as wind, biomass, solar, geothermal, and tidal power has therefore become especially important [1,2,3]. However, due to their intermittent nature, these new energy supplies cannot be used continuously. It is crucial to develop suitable equipment to convert renewable energy sources into electricity before being exported. Research into rechargeable batteries has become a worldwide research objective [4,5]. The kind of traditional nickel-cadmium, lead-acid, and nickel-metal hydride batteries are not suitable for large-scale application on account of some disadvantages such as poor energy density, short service life, and environmental pollution. In this situation, it is a main priority to develop rechargeable batteries as a replacement of these traditional batteries in the battery industry in recent decades. Secondary batteries have been considered as the most effective and dependable new-generation energy storage and conversion devices because they can freely finish energy conversion between chemical and electrical energy through reversible reactions to achieve energy storage and have a high-capacity conversion efficiency in this process [6,7,8]. Recently, there has been significant research and exploration into Li–ion batteries (LIBs) due to their many benefits, which include high energy density, extended cycling life, lightweight nature, and minimal environmental impact [9,10,11,12]. Since the 20th century, LIBs have exhibited widespread applications in smartphones, laptops, and other portable electronic devices. Currently, LIBs are used as the main power supply of electric vehicles, but they still require higher energy density and long cycling life. As the key component of LIBs, anode directly affects the voltage, capacity, and cost of the batteries [13,14,15]. Conventional anode materials of LIBs primarily contain the three categories of insertion-type (graphite, Li_4_Ti_5_O_12_, etc.), conversion-type (Fe, Co, and Ni-based metal oxides), and alloying-type (Sn, Sb, and Bi-based metal oxides) materials, which suffer from either limited capacity or frustrating cycling performance. Neither can satisfy the growing demand for higher energy density and long cycling life in LIBs. These drawbacks arise from the volume fluctuation and structural deterioration experienced by the active materials during the charge and discharge cycles, which severely limit their commercialization applications [16,17,18,19,20]. As a result, organic materials have been developed as feasible alternatives to current inorganic anode materials [21,22,23].

Organic electrode materials, particularly organic carbonyl compounds, have garnered attention due to their notable capacities resulting from the occurrence of multi-electron transfer mechanisms during electrochemical redox reactions [24,25,26]. However, the dissolution of organic materials in aprotic electrolytes can result in an undesirable and discouraging reduction in capacity over time. To address this issue, a potentially effective approach involves the implementation of organic carbonyl salts. This strategy offers the advantage of preserving the substantial capacities associated with organic components, also mitigating solubility concerns in the electrolyte through the establishment of ionic bonds. Thus far, extensive research has been accompanied with a range of organic carbonyl salts that exhibit significant theoretical capacities for use in rechargeable LIBs [27,28]. Nevertheless, solubility remains a significant concern that requires resolution. It should also be noted that the alteration in volume during the lithiation and delithiation processes plays an essential role in assessing the limits of electrode materials’ cycling stability and power density during repeated charging and discharging cycles.

Metal-organic frameworks (MOFs), constructed using the self-assembly of inorganic metal centers as nodes and organic ligands as linkers, are new generation crystalline materials with ordered networks. Owing to the features of the hybrid compositions and variable structures, inherent porous channels, and the feasible synthetic method, MOFs have been employed in various fields including catalysis, drug delivery, and energy storage and conversion [29,30]. MOFs have emerged as highly effective anode materials for LIBs in recent years [31,32]. The MOFs-based electrode materials exhibit insolubility in organic electrolytes. Furthermore, the structural flexibility of the material enables it to effectively reduce the significant volume fluctuations that occur during repeated discharging and charging cycles. As a result, this characteristic helps to overcome the degradation issues that arise due to the aforementioned volume changes. One of the primary obstacles encountered with traditional MOFs when employed as electrode materials is their low capacity for the diffusion of lithium ions, as well as the impeding effect of organic solvents on the pore window. To solve these problems, one-dimensional (1D) coordination polymers (CPs) can be utilized as electrode materials. Instead of strong covalent connections, the coordination chains are connected with weak π···π stacking interactions or hydrogen bonds. This arrangement facilitates the presence of adjustable interchain spaces, which in turn promotes rapid lithium–ion diffusion. As a result, these materials can efficiently attain their highest potential capabilities [33,34,35,36].

Among the many organic carbonyl salts, Na_2_C_5_O_5_ exhibits promising characteristics as a material with superior performance for LIBs, owing to its ability to undergo the reaction of lithium enolization at polycarbonyl oxygens [37,38]. However, during the experimental procedure, it was observed that the capability of the material was considerably limited due to its solubility in the electrolyte. A promising strategy for achieving a practical high capacity with C_5_O_5_^2−^ entails the C_5_O_5_^2−^ coordination with transition metal ions. This strategy is advantageous as the formation of coordination bonds serves to enhance the material’s stability in the electrolyte, while also facilitating the creation of specific channels for the diffusion of lithium ions [39]. This work contributes a chain-structured croconate-based Fe-CP ([Fe(C_5_O_5_)(H_2_O)_3_]_n_ (C_5_O_5_^2−^ = croconate dianion)) using a simple slow evaporation process with a little modification performed at ambient temperature. As a possible anode material for LIBs, Fe-CP was examined. The coordination between C_5_O_5_^2−^ and transition-metal ions has been discovered to have a major impact on electrolyte stability and improves the performance of the discharging/charging cycles. The CP with Fe(II) exhibited a notable 521 mA h g^−1^ specific capacity when subjected to a 100 mA g^−1^ current density following undergoing 140 cycles. Significantly, the Fe-CP demonstrated notable reversibility and remarkable rate performance along with extended cycling stability when employed as an anode material for LIBs.

## 2. Experimental Section

### 2.1. Materials and Methods

All raw reagents utilized in this study were commercially obtained and employed without undergoing additional purification procedures. Disodium croconate (Na_2_C_5_O_5_) (97 wt%) was purchased from Alfa Aesar, UK. FeSO_4_·7H_2_O (99 wt%), N-methyl-2-pyrrolidone (NMP) (99.5 wt%), and metallic lithium (99 wt%) were purchased from Shanghai Aladdin Bio-Chem Technology Co., Ltd. (Shanghai, China). Cu foils (99%) were bought from Sigma–Aldrich (Shanghai, China). Diethyl carbonate (DEC) (99 wt%) and ethylene carbonate (EC) (99 wt%) were obtained from Beijing J&K Chemical Technology Co., Ltd. (Beijing, China). The Fe-CP was synthesized using a different slow evaporation method from the study by West in 1963 [40]. The elemental analyses (EA) of hydrogen (H), carbon (C), and nitrogen (N) were conducted using a Perkin Elmer 2400-II CHNS/O analyzer. Using Labsys NETZSCH TG 209 Setaram equipment, thermogravimetric analyses (TGA) were carried out in a nitrogen environment under controlled conditions. The analysis was accomplished at a 10 °C min^−1^ heating rate at temperatures ranging from 40 to 800 °C. Powder XRD (PXRD) patterns were acquired employing Rigaku Ultima IV equipment and Cu-Kα radiation between 3 and 50° 2*θ*. The scanning rate employed for these measurements was 5° min^−1^. Fourier transform infrared (FTIR) spectra were gathered between 400 and 4000 cm^−1^ utilizing a Bruker ALPHA-T infrared spectrophotometer. A ZEISS MERLIN Compact (Field Emission) scanning electron microscope (SEM) was employed to obtain the morphologies. A Kratos AXIS Ultra DLD spectrometer equipped with an Al-Kα X-ray source was employed to perform X-ray photoelectron spectroscopy (XPS).

### 2.2. Electrochemical Analyses

To assess the electrochemical properties of the Fe-CP material, 2032 coin cells were created. Electrode active materials, polyvinylidene fluoride (PVDF) and Ketjet Black, were uniformly dispersed in N-methyl-2-pyrrolidone (NMP) at respective wt% of 70, 10, and 20 to produce a slurry. After applying the slurry to the copper foil, it was dried for 12 h at 80 °C. Following the drying process, the material was punched into discs with a 12 mm diameter which was then subsequently utilized as an electrode. The loading of the active material was approximately 1.0 mg cm^−2^. In the coin cells’ construction process, a Li was employed as both the reference and counter electrode. LiPF_6_ solution (1 M) in a 1:1 *v*/*v* mixture of diethyl carbonate and ethylene carbonate (DEC/EC) served as the electrolyte. Similarly, polypropylene film was utilized as the separator. The procedure was conducted within a glovebox filled with argon gas, ensuring that the levels of oxygen and water were maintained below 0.1 ppm. Then, the batteries were aged 12 h. The cells were subjected to electrochemical impedance spectroscopy (EIS) and cyclic voltammetry (CV) using a PARSTAT 4000 electrochemical workstation. A LAND battery station was subjected to galvanostatic charge/discharge measurements between 0.01 and 2.4 V (vs. Li/Li^+^) at 298 K.

### 2.3. Synthesis of the Fe-CP

Fe-CP was synthesized using a simple slow evaporation method. A total of 27.8 mg of FeSO_4_·7H_2_O (0.1 mmol) along with 9.3 mg of disodium croconate (Na_2_C_5_O_5_, 0.05 mmol) were dissolved in 2 mL of deionized water, respectively. Subsequently, the two solutions were mixed and allowed to slowly evaporate at ambient temperature. Dark brown crystals of the Fe-CP were produced after three days. The collected crystals were thoroughly washed with water and ethanol three times to eliminate any impurities. The product was acquired with a yield of 72%, calculated with respect to Na_2_C_5_O_5_. Anal. Calcd. for C_5_H_6_FeO_8_: C, 24.03; H, 2.42. Found: C, 24.07; H, 2.45. 

## 3. Results and Discussion

**Figure 1:**
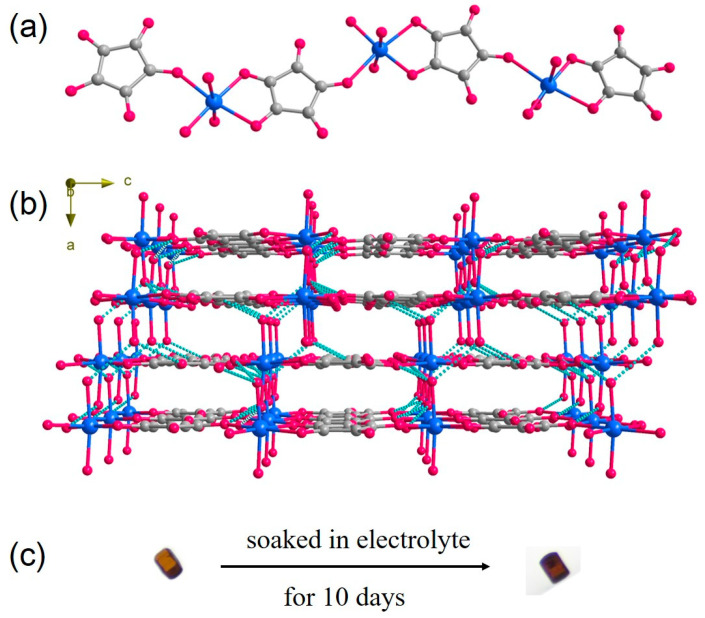
(**a**) The 1D structure of Fe-CP. Red, gray, and blue balls represent O, C atoms, and metal, respectively. (**b**) Supramolecular networks are built by forming hydrogen bonds between the chains. The dotted green lines denote hydrogen bonding (O-H···O). (**c**) The 10-day dissolution test in electrolyte exhibits the remarkable stability of Fe-CP in the electrolyte.

The synthesis of Fe-CP was initially conducted by West in 1963, followed by the subsequent determination of its structure by Cornia [41]. The Fe-CP exhibited crystallization in the orthorhombic space group *Pbca*. In this structure, each Fe^2+^ ion was coordinated using six atoms, consisting of three O atoms from two C_5_O_5_^2−^ ligands and three H_2_O molecules. The Fe^2+^ centers were coordinated in an alternating fashion to create a polymeric structure with an infinite chain (Figure 1a). The adjacent chains were then joined to construct a three-dimensional supramolecular structure via interchain O-H···O hydrogen bonds (Figure 1b). Hydrogen bond weakly-linked coordination chains facilitate the modulation of inter-chain spacing, thereby facilitating efficient diffusion of Li^+^ ions and enabling the material to reach its greatest potential capacities. In the organic electrolyte, the stability of Fe-CP was significantly enhanced and was unsolvable in the electrolyte through the formation of supramolecular networks that are primarily composed of coordination chains. Figure 1c shows that, after soaking the Fe-CP sample in the electrolyte for 10 days, transparent single crystals were produced and were similar in appearance to the pure ones. Moreover, PXRD patterns of the Fe-CP sample were examined both before and after immersion in the organic electrolyte, providing further evidence of the Fe-CP’s stability (Figure S1).

The confirmation and characterization of the structure and thermostability of the Fe-CP were conducted using techniques such as PXRD, FTIR, and TGA. The Fe-CP material exhibited a high degree of crystallinity and demonstrated excellent thermal stability. The experimental PXRD pattern of Fe-CP exhibited a strong agreement with the simulated pattern derived from the crystal structure (Figure S2). According to the TGA curve, between 120 and 280 °C, the sample lost 21.59% of its weight due to the loss of three coordinated water molecules (Calcd. 21.62%). The framework’s structural integrity did not begin to deteriorate until it reached 340 °C (Figure S3). The stretching vibrations of the hydroxyl groups in the water molecules cause a broad and noticeable peak in the FTIR spectra between 3000 and 3400 cm^−1^ (Figure S4). Furthermore, the prominent peak for the *ν*_HOH_ bending vibrations appeared at 1627 cm^−1^. The absorption peak for the non-coordinated C=O group appeared at 1723 cm^−1^, while the peak corresponding to the C-O group in coordination appeared at 1677 cm^−1^. The absorption peak between 1300 and 1600 cm^−1^ exhibited a wide and strong intensity, indicating the presence of the C_5_O_5_^2−^ anion [42]. This peak can be attributed to a combination of C-C and C-O stretching vibrational modes. Furthermore, it was noted that Fe-CP displayed multiple absorptions within the range of 400 to 570 cm^−1^. These absorptions can be assigned to the *ν*_M-O_ stretching vibrations, indicating the generation of coordination bonds between C_5_O_5_^2−^ anions and Fe^2+^ ions [43]. 

**Figure 2:**
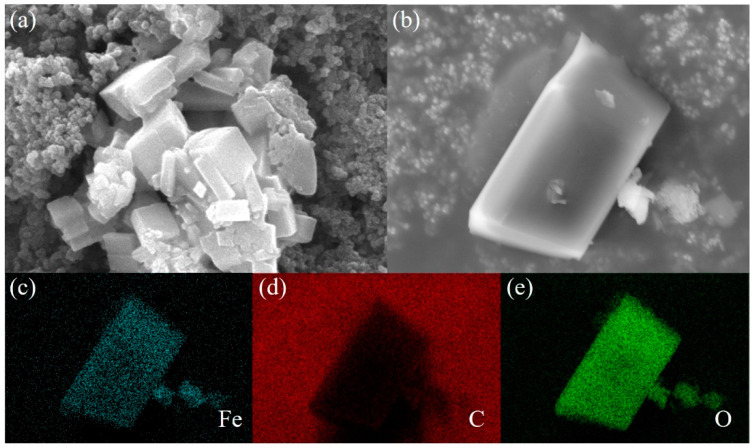
(**a**,**b**) SEM images of Fe-CP. (**c**–**e**) EDS mapping images of Fe, C, and O of Fe-CP.

SEM measurements were conducted to study the morphology of the Fe-CP. As shown in Figure 2a,b, Fe-CP exhibited a regular shape, displaying a well-defined lump structure. Furthermore, the EDS mapping (Figure 2c–e) was performed to confirm the elemental distribution of the synthesized products, revealing a uniform distribution of Fe (in blue), C (in red), and O (in green). The homogeneous distribution of Fe elements enabled the polymer to possess a greater number of electrochemically active sites to afford high capacity.

**Figure 3:**
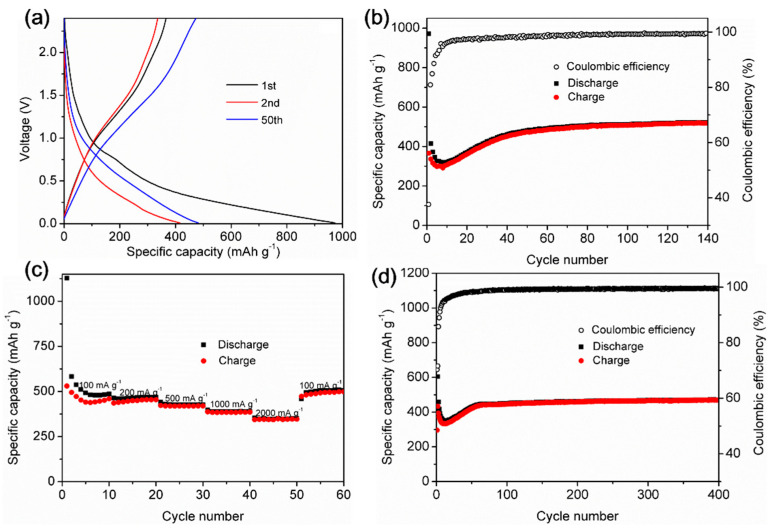
(**a**) GCD curves at 100 mA g^−1^. (**b**) Coulombic and cycling efficiencies during 140 cycles at 100 mA g^−1^. (**c**) Results of rates at various current densities. (**d**) Cycling efficiency at 500 mA g^−1^.

Using a two-electrode 2032 coin-cell battery, the Fe-CP were first tested for their capacity to store Li-ion. Li served as the counter electrode, having a voltage range of 0.01 to 2.4 V and a current density of 100 mA g^−1^. The initial discharge capacity of the Fe-CP anode was 972 mA h g^−1^ in the first cycle, indicated in the galvanostatic charge/discharge (GCD) profile (Figure 3a). In the second cycle, it dropped to 415 mA h g^−1^. The initial capacity loss is mainly caused by the formation of a solid electrolyte interphase (SEI) layer and interfacial lithium storage. In addition, the reaction of coordinated water molecules within the Fe-CP anode with lithium to form Li_2_O is also responsible for the first cycle’s irreversible capacity loss [44,45]. However, the anode exhibited consistent reversible Li-ion uptake, thereafter accompanied with stable SEI film formation and the disappearance of side reactions. This phenomenon is commonly observed in various anode materials. After being cycled at 100 mA g^−1^, the Fe-CP anode showed a final capacity of 521 mA h g^−1^. After being subjected to 140 cycles, the anode still managed to preserve 99.3% of its original capacity (Figure 3b). The presence of symmetrical voltage profiles in the GCD curves, along with nearly 100% Coulombic efficiency throughout cycling, implies that the Fe-CP anode has a robust structure during the lithiation/delithiation processes, giving rise to a high-level of cycle stability. To further investigate the cycling stabilities and rate capacities, we studied the rate performance of the Fe-CP anode. Discharge and charge cycles at various current densities (from 100 to 2000 mA g^−1^) are shown in Figure 3c. At 100, 200, 500, 1000, and 2000 mA g^−1^, Fe-CP had specific discharge capacities of 460, 455, 420, 386, and 347 mA h g^−1^, respectively. When the current rate reverted to 100 mA g^−1^, the electrode exhibited a restoration of its initial capacity, suggesting that the anode material maintained its stability throughout the process of rate cycling. The cycling performances of Fe-CP were studied at higher current densities to further assess its lithium storage capability. It was found that the Fe-CP anode, when exposed to a higher current density of 500 mA g^−1^, maintained a constant capacity of 471 mA h g^−1^ throughout 400 cycles, which is much higher than the theoretical capacity of graphite (372 mA h g^−1^). Furthermore, the anode’s Coulombic efficiency was nearly 100% (Figure 3d). We also investigated the fabricated Fe-CP anode after 5 days for comparison. As shown in Figure S5, the initial discharge capacity of the Fe-CP anode was 1014 mA h g^−1^ in the first cycle, and it remained at a capacity of 517 mA h g^−1^ with a 98.1% Coulombic efficiency after 26 cycles. The capacity and Coulombic efficiency from the 26th cycle onward tended to remain nearly constant, indicating the good stability and capacity retention of the Fe-CP anode after standing for 5 days. On the basis of the above results, it can be stated that the Fe-CP contains competitive high-performance anode materials in comparison to other typical anode materials in LIBs (Table 1) [46,47,48,49,50,51,52,53,54,55,56,57,58,59,60,61,62,63,64,65,66,67,68,69,70]. These findings highlight the exceptional potential of 1D structure materials for lithium storage, offering enhanced performance and reliability for LIBs.

**Figure 4:**
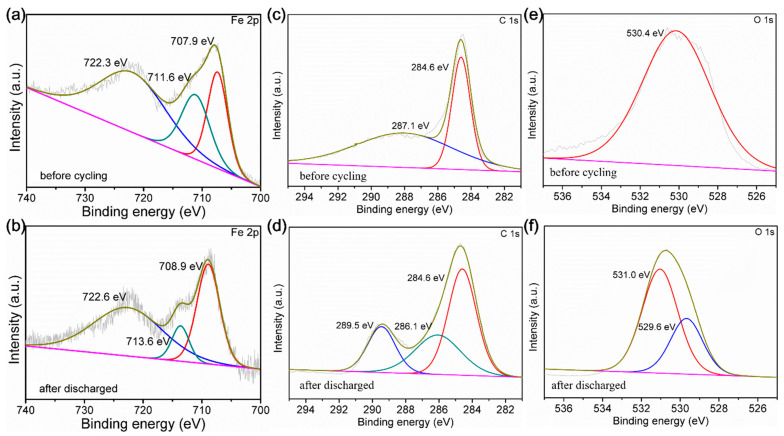
XPS spectra (**a**,**b**) of Fe 2p before and after discharge. Spectra (**c**,**d**) of C 1s of Fe-CP from constructed electrodes before and after electrochemical discharge procedures. Spectra (**e**,**f**) of O 1s before and after discharge.

Ex situ XPS measurements were utilized to investigate the mechanism of the redox reaction in the Li-ion battery. The XPS analysis of the Fe 2p spectrum following complete discharge (at 708.9 and 722.6 eV) exhibited similar peak positions to those which appeared in a pristine state (at 707.9 and 722.3 eV). This suggests that there was no reduction of Fe^2+^ ions during the discharge process (Figure 4a,b) [71]. As depicted in Figure 4c,d, the C 1s spectra obtained after discharge showed three distinct peaks at energies of 284.6, 286.1, and 289.5 eV for Fe-CP. These peaks correspond to the C=C bonds (of the five-membered ring), C-C bonds (of carbonyl groups), and enol structures [72]. The conversion of carbonyl into enol after complete discharge provides evidence for the mechanism of electron absorption with organic functional groups. During the discharging process, the O 1s spectra showed two peaks (at 529.6 and 531.0 eV), which were attributed to lattice oxygen and the Li-O bond, respectively (Figure 4e,f) [72]. Additionally, CV tests in the voltage range (from 0.01 to 3 V) and scan rate (0.1 mV s^−1^) were carried out to examine the Li insertion/extraction process of Fe-CP. Figure S6 depicts the results of an initial investigation of CV curves, which demonstrated clear irreversibility as a result of SEI layer generation. This phenomenon was a consequence of irreversible reactions between the electrode and the electrolyte, as well as the irreversible lithiation reaction involving water molecules. The CV curves observed in the subsequent scans exhibited a high degree of overlap, suggesting that the SEI layers formed during the initial scan exhibit exceptional stability, and the electrode reactions demonstrate a high level of reversibility. This observation suggested that Fe-CP would demonstrate excellent cycling performance with minimal side reactions involving the electrolyte. Only one pair of redox peaks was observed, suggesting the occurrence of a single redox reaction process throughout the discharge/charge cycles. Following the initial scan, a single oxidation peak was detected at 0.97 V, indicating the exclusive involvement of the organic moieties in Li storage. Moreover, no reduction of the Fe^2+^ center occurred during the process of Li insertion and extraction [73].

This study also analyzed the anode’s EIS in its original state and after 140 discharge/charge cycles to gain a more in-depth understanding of the reasons contributing to the Fe-CP’s outstanding electrochemical characteristics. According to the data presented in Figure S7, the diameter of the semicircle associated with the Fe-CP anode experienced a notable reduction after 140 cycles. This observation suggests an enhancement in the Li^+^ diffusion rate and electron transport during the cycling process. Such improvements are highly advantageous for achieving notable rate capabilities and maintaining excellent cycling performance [74]. These results further support the previous findings of excellent cycling stability and rate capability, highlighting the potential of Fe-CP as a promising anode material for LIBs.

**Figure 5:**
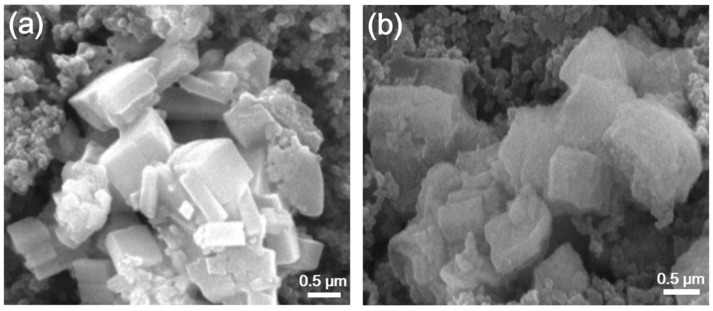
SEM images of Fe-CP as electrode before cycling (**a**) and after 40 cycles (**b**).

Electrode materials must be able to maintain their cycling stability during the charging and discharging cycles. To ascertain the Fe-CP anode’s structural stability, the electrode-containing coin cells were disassembled after undergoing 40 cycles. Subsequently, electrode discs were utilized to extract the active materials and were subjected to multiple washes utilizing dimethyl carbonate (DMC). Based on the comprehensive analysis of PXRD, FTIR, and SEM measurements, it can be inferred that the Fe-CP material exhibits remarkable durability when Li+ ions are repeatedly inserted and extracted. When the electrode materials were studied using FTIR spectroscopy and PXRD in their fully charged condition (2.4 V), they showed excellent agreement with the original Fe-CP material (Figures S8 and S9). Furthermore, Figure 5 displays the results, showing that the Fe-CP sample maintains its original morphology even after 40 discharge/charge cycles. This observation suggests that the supramolecular structures formed with the chains in the Fe-CP material are capable of accommodating the significant volume changes that occur during the delithiation/lithiation processes. Moreover, Figure 3b,d provide clear evidence that the capacity and Coulombic efficiency exhibit a constant pattern from the 40th cycle onwards. This suggests that there are no observable alterations in the morphology and crystalline structure of the samples after undergoing extensive cycling, up to 140 and even 400 cycles.

## 4. Conclusions

In conclusion, this study focuses on the investigation of a 1D croconate-based Fe-CP as a potential anode material with enhanced performance for LIBs. The Fe-CP anode exhibited reduced solubility in the electrolyte and was not subject to the limitations in channel size for enhanced Li^+^ diffusion, thereby leading to a significant increase in the specific capacities of the anode materials. The electrochemical mechanism studies revealed that the Li insertion/extraction process is largely influenced by the synergistic redox reactions of organic C_5_O_5_^2−^ moieties, high structural stability, and low resistance to charge transfer. In the context of the anode materials, the Fe-CP anode exhibited a notable 521 mA h g^−1^ reversible capacity in half-cells after 140 cycles. In addition, after 400 cycles, a stable 471 mA h g^−1^ lithiation capacity was attained at 500 mA g^−1^, verifying the endurance of Li^+^ insertion and extraction processes and the cycling stability of Fe-CP over the long term. Fe-CP was found to have a high capacity and increased cycling stability, making it an effective anode material for LIBs.

## Figures and Tables

**Table 1:** Performances of Fe-CP and some other typical anode materials in LIBs.

Type of Anode Material	Typical Examples	Current Density (mA g^−1^)	Capacity (mA h g^−1^)	Cycle Number	Ref.
Conventional inorganic materials	Multichannel graphite	0.1C	365	3000	[46]
	Expanded graphite	100	338	500	[47]
	Al_2_O_3_@graphite	100	335	100	[48]
	P-doped mesoporous C	0.5C	500	200	[49]
	Li_5_Cr_7_Ti_6_O_25_@CeO_2_	5C	100.5	100	[50]
	Ti_2_Nb_10_O_29_/C	10C	194	100	[51]
	Fe_2_O_3_ nanotubes on 3DG aerogel	100	1406	65	[52]
	Co_3_O_4_@C	250	721	500	[53]
	ZnO@C	250	526	500	[53]
	Zn_2_SiO_4_ nanowires	100	455.8	100	[54]
	Zn_2_SiO_4_@NC nanowire	100	685.2	100	[54]
Organic anode materials	LiTA	100	133.5	100	[55]
	CMH	200	404.6	250	[56]
	NBALS	0.5C	153	100	[57]
	Li_2_-DBT	0.1C	122	50	[58]
	Li_2_-DMoT	0.1C	95	50	[58]
	Li_2_-DAT	0.1C	98	50	[58]
	E-CIN-1/CNT	100	1005	250	[59]
	E-SNW-1/CNT	100	920	250	[59]
	PA-TA	1000	543	400	[60]
	rCTF	300	1190	1000	[61]
CP-based anode materials	Mn(2,5-FDC)·3H_2_O	300	503	326	[62]
	Mn(3,5-PDC)·2H_2_O	100	554	240	[62]
	Co-LCP	50	545	50	[63]
	Mn-BTC	100	845	100	[64]
	[Cd(HTCPPA)·2H_2_O]_n_	100	302	100	[65]
	Cu_3_(BTC)_2_	383	474	50	[66]
	Co-BDC	100	1090	1000	[67]
	[Pb(4,4′-ocppy)_2_]·7H_2_O	100	489	500	[68]
	Mn-3D	100	692	160	[69]
	Co-MOF	100	400	80	[70]
	Fe-CP	100	521	140	This work

## Data Availability

Not applicable.

## Supplementary Materials

The following supporting information can be downloaded at: https://www.mdpi.com/article/10.3390/polym15183728/s1. Figure S1: PXRD of Fe-CP after soaked in electrolyte for 10 days; Figures S2–S4: PXRD, TGA and FTIR of the as-synthesized Fe-CP; Figure S5: Coulombic and cycling efficiencies of the fabricated Fe-CP anode after standing for 5 days; Figure S6: Cyclic voltammetry curves of the 1st and subsequent cycles in the range of 0.01–2.4 V at a scan rate of 0.1 mV s^−1^; Figure S7: Impedance plots of Fe-CP as anodes before and after 140 cycles; Figures S8 and S9: PXRD and FTIR of Fe-CP before and after 40 cycles.
