# Supplementary material for: One-Dimensional Croconate-Based Fe-CP as a High-Performance Anode Material for Lithium–Ion Batteries
Source: Polymers (Basel). 2023 Sep 11;15(18):3728. doi: 10.3390/polym15183728 (PMC10535239; doi:10.3390/polym15183728)
Supplement: Supplementary file 1 [file polymers-15-03728-s001.zip › polymers-2561865-supplementary.pdf]

Supporting Information

One-dimensional Croconate-based Fe-CP as a High Performance Anode Material for Lithium Ion Batteries

Lin Zhang,* Xiaofei Zhang and Yingcai Gui

Henan Key Laboratory of Functional Salt Materials, Center for Advanced Materials Research, Zhongyuan University of Technology, Zhengzhou 450007 (P. R. China).

Email: 6695@zut.edu.cn.

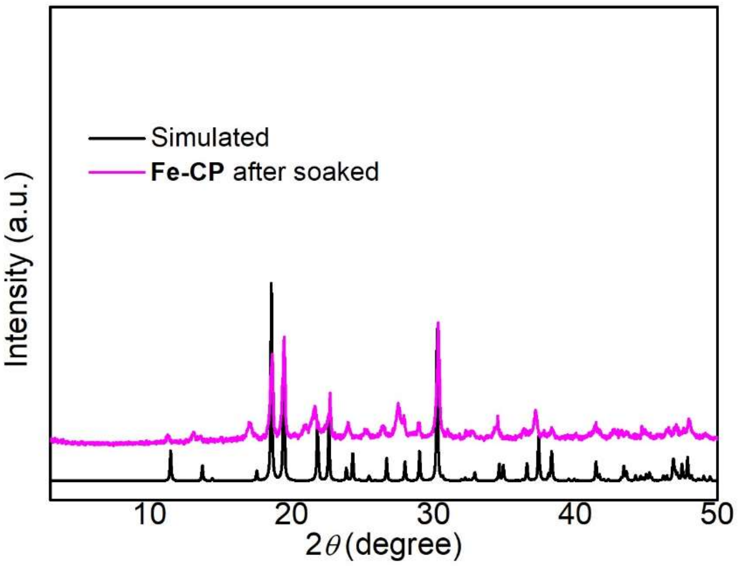

Figure S1. Powder X-ray diffraction patterns (PXRD) of **Fe-CP** after soaked in electrolyte for 10 days.

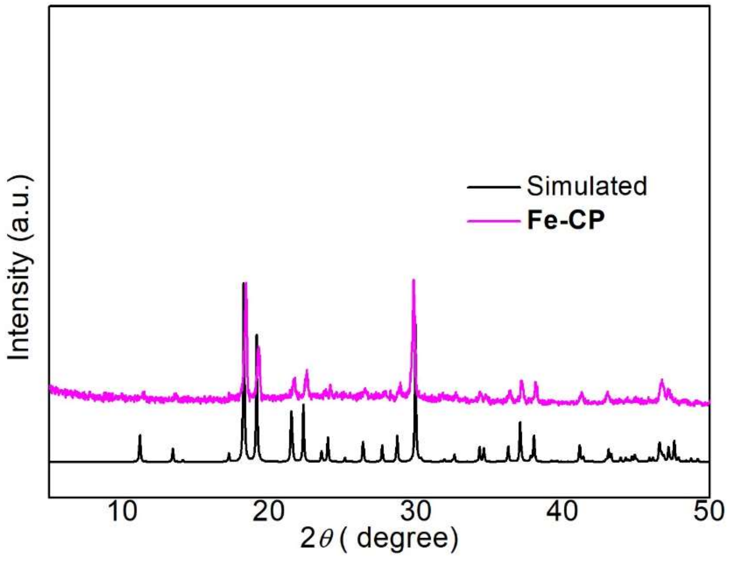

Figure S2. Simulated and experimental PXRD patterns of **Fe-CP**.

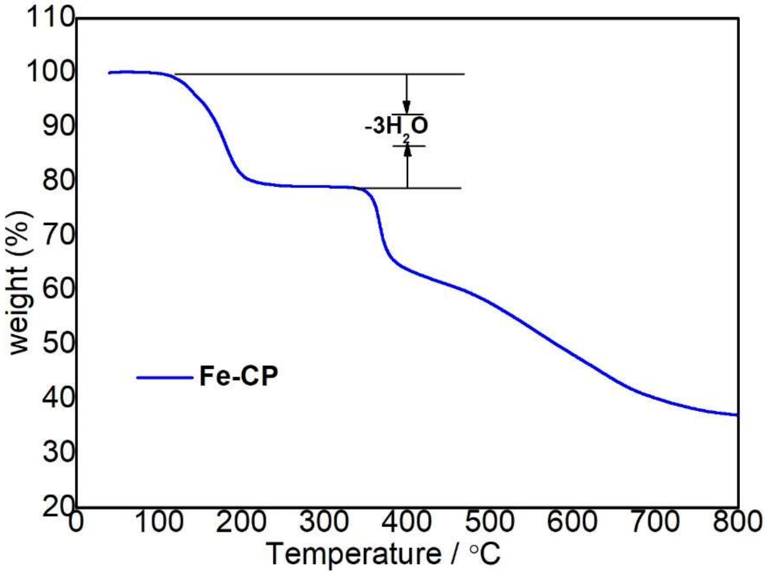

Figure S3. Thermal gravimetric analysis (TGA) curves.

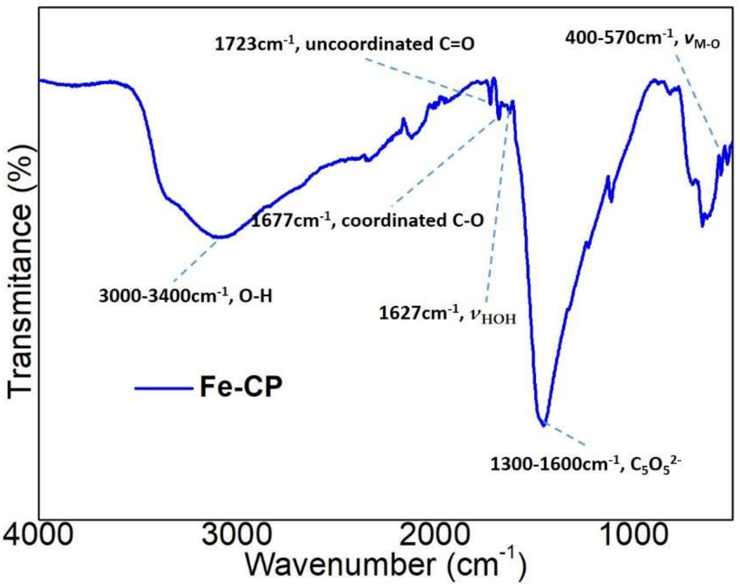

Figure S4. FTIR of the as-synthesised **Fe-CP**.

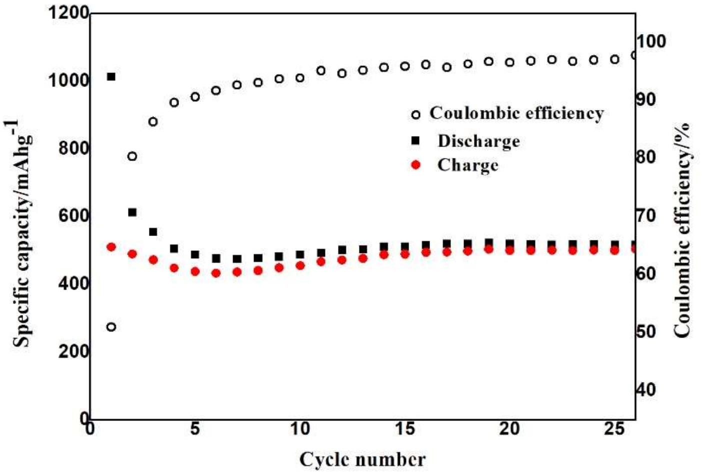

Figure S5. Coulombic and cycling efficiencies of the fabricated **Fe-CP** anode after standing for 5 days.

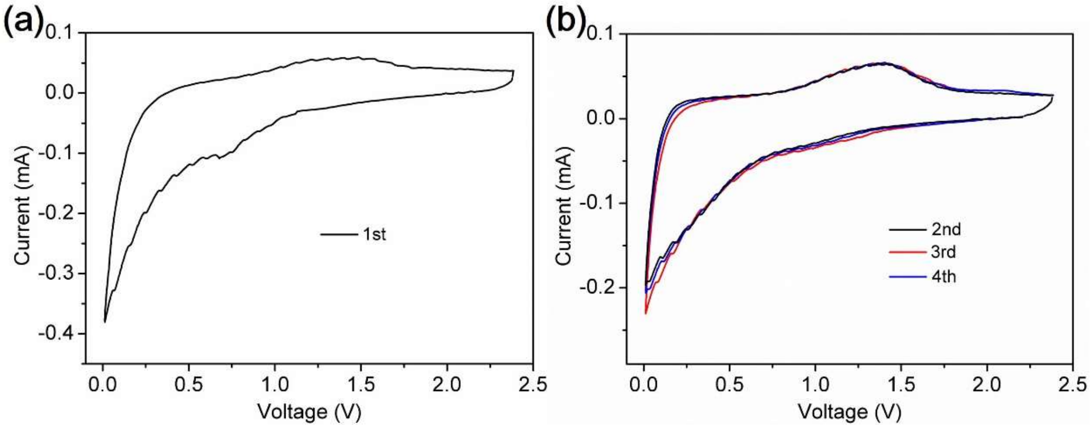

Figure S6. Cyclic voltammetry curves of the 1st and subsequent cycles in the range of 0.01–2.4 V at a scan rate of 0.1 mV s^{-1} for **Fe-CP**.

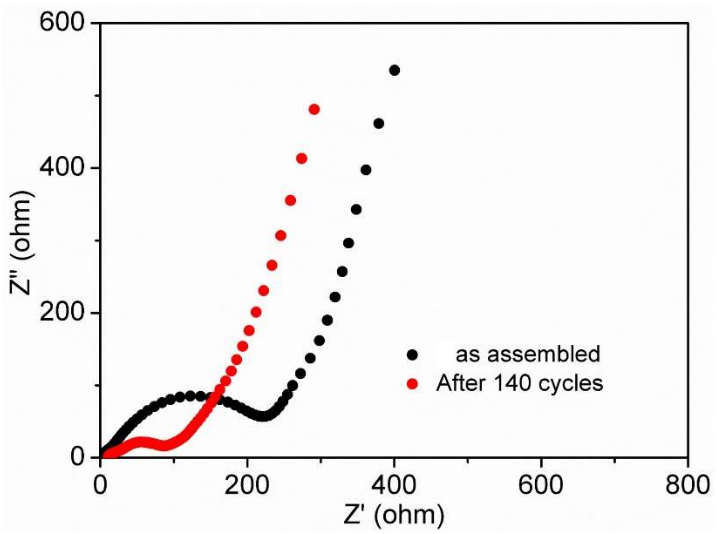

Figure S7. Impedance plots of **Fe-CP** as anodes before and after 140 cycles.

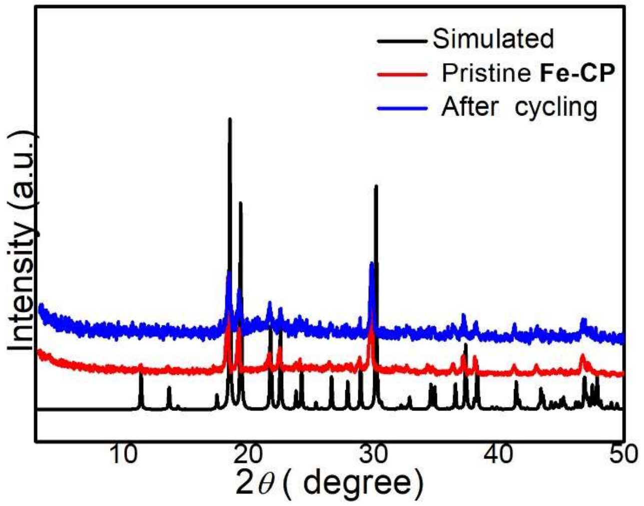

Figure S8. PXRD patterns of **Fe-CP** before and after 40 cycles.

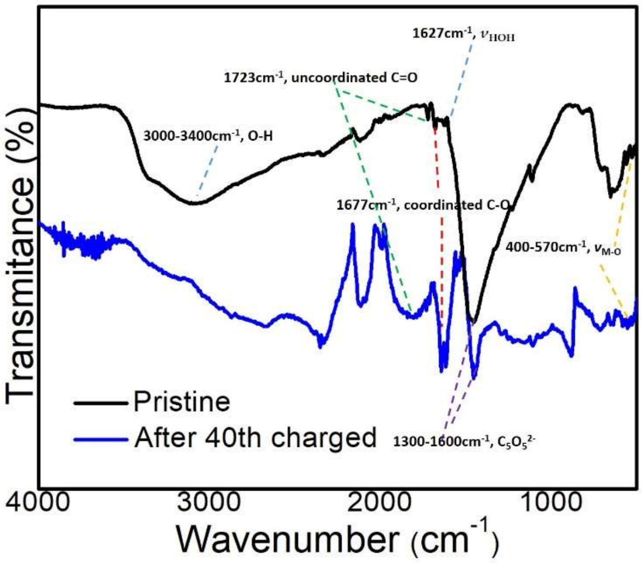

Figure S9. FTIR before and after 40 cycles.
